# Loss of mdig expression enhances DNA and histone methylation and metastasis of aggressive breast cancer

**DOI:** 10.1038/s41392-018-0027-4

**Published:** 2018-09-21

**Authors:** Chitra Thakur, Bailing Chen, Lingzhi Li, Qian Zhang, Zeng-Quan Yang, Fei Chen

**Affiliations:** 1 Department of Pharmaceutical Sciences, Eugene Applebaum College of Pharmacy and Health Sciences, Wayne State University, Detroit, MI 48201 USA; 2 Synthesis Medchem Corp, 425 Changyang Street, Suzhou Industrial Park, Suzhou, 215025 China; 3 City of Hope Institute, 1500 E. Duarte Road, Duarte, CA 91010 USA; 4 Department of Oncology and Barbara Ann Karmanos Cancer Institute, Wayne State University, Detroit, MI 48202 USA

## Abstract

We previously reported that expression of an environmentally induced gene, mineral dust-induced gene (*mdig*), predicts overall survival in breast cancer patients. In the present report, we further demonstrate the differential roles of mdig between earlier- and later-stage breast cancers. In noncancerous breast, mdig is a proliferation factor for cell growth and cell motility. In breast cancer, however, higher levels of mdig negatively regulate the migration and invasion of cancer cells. Assessment of global DNA methylation, chromatin accessibility and H3K9me3 heterochromatin signature suggests that silencing mdig enhances DNA and histone methylation. Through immunostaining and data mining, we found that mdig is significantly upregulated in noninvasive and/or earlier-stage breast cancers. In contrast, in triple-negative and other invasive breast cancers, diminished mdig expression was noted, indicating that the loss of mdig expression could be an important feature of aggressive breast cancers. Taken together, our data suggest that mdig is a new biomarker that likely promotes tumor growth in the early stages of breast cancer while acting as a tumor suppressor to inhibit invasion and metastasis in later-stage tumors.

## Breast cancer: Gene expression influences tumor development

Differential expression of an environmentally-induced gene appears to influence the growth of breast cancer tumors, thus providing a valuable biomarker and therapeutic target. Environmental factors can influence cancerous tumor development by interfering with epigenetic processes such as DNA and histone methylation. For example, the mineral dust induced gene (mdig) is over-expressed in coal miners who are susceptible to lung cancer. Now, Fei Chen, a pioneer in toxicology and carcinogenesis research at the Wayne State University in Detroit, USA, and his team have demonstrated that mdig also plays important roles in breast cancer. The gene is upregulated in early, non-invasive tumors, where it regulates cell growth, motility and invasion by influencing DNA and histone methylation. However, mdig expression drops in later stage or more aggressive tumor types. When the researchers abrogated mdig expression completely, they observed an enhanced DNA and histone methylation, suggesting the gene has a demethylase role and is implicated in regulating the epigenetic landscape under neoplastic conditions.

## Introduction

Breast cancer is the second-most common and the leading cause of cancer deaths in women in the USA. In addition to certain genetic predispositions, such as mutations in the BRCA1 and BRCA2 genes, a number of environmental risk factors, including chemical carcinogens, ionizing radiation, toxic metals, tobacco smoke, and alcohol consumption, have been considered potential etiologic factors for breast cancer through their actions on estrogenic activity or other important intracellular signaling pathways. The mineral dust-induced gene (mdig) was first discovered in alveolar macrophages from coal miners who had been exposed to mineral dust under occupational settings, and it is a lung cancer-associated oncogene.^1^ Independently, this gene was also identified in human promyelocytic leukemia HL60 cells and human glioblastoma cell line T98G, and named myc-induced nuclear antigen 53 (mina53).^2^ Several studies have reported increased expression of mdig in a number of human cancers, particularly cancers of the lung and breast.^3^ The mdig protein has 465 amino acids with a conserved JmjC domain, a signature motif in the majority of histone demethylases.^4^ Emerging evidence suggests a role for mdig in cell proliferation, neoplasias,^3^ pulmonary inflammation^5,6^ and immune regulation.^7,8^ Intriguingly, we previously observed a paradoxical role of mdig in cell proliferation, migration and invasion in cellular experiments.^9^ Some environmental factors, including silica, arsenic, and tobacco smoke, induce the expression of mdig, possibly through JNK-STAT3 signaling.^10^

Both genetic and epigenetic changes are integral to the complex process of breast carcinogenesis. Epigenetic alterations, such as DNA methylation, posttranslational modification of histones, microRNAs, and long-noncoding RNAs, are gaining wide acceptance in the field of cancer as contributors to cancer biology. DNA methylation is the most commonly studied epigenetic modification in cancer, which comprises the addition of a methyl group onto the fifth carbon of the cytosine within or outside of the CpG island.^11^ During the process of carcinogenesis, it is believed that the DNA of some tumor suppressor genes, such as HOXA5, TMS1, p16, RASSF1A, and BRCA1, are hypermethylated and silenced due to abnormal expression or activity of DNA methyltransferases (DNMTs).^12–14^

In this report, we provide evidence showing that mdig regulates cell growth, breast cancer cell migration and invasion partially through DNA, as well as histone methylation. In both in vitro experiments and analysis of tissue samples from breast cancer patients, we found that levels of mdig expression are negatively correlated with DNA methylation, cell migration and invasion. Silencing mdig increased the invasion and migration potential of breast cancer cells, as well as elevated the mRNA levels of genes involved in invasion and motility. Analysis of human breast cancer samples and breast cancer databases revealed that the role of mdig in the pathogenesis and prognosis of breast cancer is context-dependent. Levels of mdig are higher in noninvasive breast cancers than in invasive breast cancers. In aggressive breast cancer, such as invasive ductal carcinoma (IDC) and triple-negative breast cancer (TNBC), mdig expression is significantly downregulated. Taken together, these data strongly suggest that the oncogenic role of mdig may be dependent on the progression stage in breast cancer. It is very likely that in earlier stages of breast cancer development, mdig is oncogenic and promotes the growth of tumor cells, whereas it may be inhibitory to the metastasis of cancer cells in later stages.

## Materials and methods

### Cell culture

The human normal breast cell line MCF 10 A and breast cancer cell lines MCF-7, MDA-MB-231, T-47D, ZR-75-1, HCC 1187 and HCC 1954 were purchased from American Type Culture Collection (Manassas, VA). MCF10A cells were cultured in Ham’s F-12 medium with 5% FBS, supplemented with insulin, hydrocortisone, EGF, ethanolamine, HEPES, transferrin, triiodo thyronine (T3), sodium selenite, and ovine serum albumin. MCF-7 and T-47D cells were cultured in DMEM, MDA-MB-231 in DMEM F-12, and ZR-75-1, HCC 1187 and HCC 1954 cells were cultured in RPMI 1640 medium. All cells were supplemented with 10% FBS and 1% penicillin-streptomycin (Sigma, St. Louis MO) and grown in 37 °C-humidified incubators in the presence of 5% CO_2._

### siRNA transfection

Reverse transfections were performed on 5 × 10^5^ cells using Lipofectamine RNAiMAX (Invitrogen) according to the manufacturer’s protocol. Fifty-nanomolar siRNA was used for transfections. Cells were cultured 24 h for gene silencing. Control siRNA and mdig siRNAs were purchased from Qiagen (Valencia, CA, USA).

### RT-PCR

Total RNA was prepared by lysing cells with TRIzol reagent (Life Technologies, Grand Island, NY, USA) according to the manufacturer’s protocol, and their integrity was assessed using 18 S and 28 S ribosomal RNAs. Reverse transcription and PCR were performed using the Access Quick RT-PCR System (Promega, Madison, WI) with 1 µg total RNA and 0.3 µM each of forward and reverse primers. Mdig primers amplified a 1,509 bp cDNA fragment covering the entire coding region of mdig mRNA. GAPDH was used as a loading control. Primer sequences for mdig are as follows: left primer, 5′-TCATGTCGGGCCTAAGAGAC-3′; and right primer, 5′GGCATTTGATTCTGCAAAGG-3′. Primer sequences for GAPDH are as follows: left primer, 5′-CTGAACGGGAAGCTCACTGGCATGGCCT-3′; and right primer, 5′ CATGAGGTCCACCACCCTGTTGCTGTAG-3′. PCR products were run on 0.8% agarose gels. mRNAs for other genes were assessed by real-time PCR using the LightCycler^®^ 480 SYBR Green I Master (Roche Diagnostics, Indianapolis) in a LightCycler^®^ 480 II PCR platform from Roche. Reaction mixtures for target genes, as well as the reference gene GAPDH, were set up in 20 μl final volume containing 3 μl cDNA (1:10 dilution), forward and reverse primers (0.3 μM), 10 μl of SYBR Green I Master mix and 5 μl nuclease-free water. Thermal cycling for genes comprised an initial activation at 95 °C for 5 min, followed by amplification that included denaturation at 95 °C for 10 s, annealing at 60 °C for 10 s, and extension at 72 °C for 10 s for 45 cycles. Each gene was tested and optimized for optimum annealing temperature. Samples without cDNA or RT templates served as negative controls. Relative mRNA expression levels of target genes were calculated with respect to the housekeeping gene GAPDH, according to the LightCycler^®^ 480 II Relative Quantification software instructions based on the 2^-ΔΔCT^ method.

### Transwell migration and invasion assay

Twenty-four well plates, 8.0-μm pore membranes, transwell and Matrigel invasion chambers (Corning USA) were used according to the manufacturer’s protocol. First, chambers were rehydrated with serum-free medium for 2 h at 37 ^°^C. Each upper chamber was supplied with 600 µl serum-free medium containing 5 × 10^4^ cells for migration and 1 × 10^5^ cells/well for the invasion assay. Next, 100 µl transfection mixture was added. Simultaneously, 250 µl cell culture medium with 5% FBS (without antibiotics) was added to the lower chamber as a chemoattractant, and cells were incubated for 24 h at 37 °C. The inserts (upper chambers) were removed, and the nonmigrated and noninvaded cells remaining on the upper surface of the membrane were scrapped off using cotton swabs. The inserts were then stained with Diff-Quick stain kit (Dade Behring Inc., Newark, DE) according to the manufacturer’s instructions. Inserts were dried and imaged under a bright field microscope. Images were captured at ×10 magnification for five different fields and were counted using ImageJ software (National Institute of Health, Bethesda, USA) (https://imagej.nih.gov/ij/).

### Cell proliferation assay

Cell proliferation was determined with the 3-(4,5-dimethylthiazol-2-yl)-2,5-diphenyltetrazolium bromide (MTT) assay using the Cell Proliferation Kit I (Roche Diagnostics Indianapolis) according to the manufacturer’s instructions. Each well was supplied with 5 × 10^3^ cells in 500 µl medium in 96-well plates. At indicated time points after initial seeding, MTT assays were performed, and optical density (OD) was measured at 570 nm using a Biokinetics plate reader (Bio-Tek Instruments, Inc, Winooski, VT, USA). Each experiment was repeated three times for reproducibility.

### DNA methylation assay

Colorimetric quantification of global DNA methylation was performed using the Methylated DNA Quantification Kit (ab117128, Abcam, Cambridge, MA) and Hydroxymethylated DNA Quantification Kit (ab117130, Abcam Cambridge, MA) according to manufacturer’s protocol. Genomic DNA was isolated from noncancerous breast cells and breast cancer cells transfected with siRNAs for mdig and control siRNA. Isolated DNA was quantified using NanoDrop™, and input DNA was diluted in TE buffer to an optimum 100 ng per reaction. Single-point positive control assays were applied, and the absorbance was read at 450 nm. Relative quantification of 5-mC was calculated using the formula 5-mC % = [(sample OD−negative control OD) ÷ S/(positive control OD−negative control OD) × 2 ÷ P] × 100, where S is the amount of input sample DNA in ng, P is the amount of input positive control in ng, and 2 is a factor to normalize 5-mC in the positive control to 100%, as the positive control contains only 50% of 5-mC. For analyzing hydroxylated DNA, the input DNA was 200 ng per reaction. The single-point positive control assay procedure was utilized, and relative quantification of 5-hmC was calculated using the formula 5-hmC % = (Sample OD – Negative Control II OD) ÷ S/(Positive Control OD−Negative Control II OD) × 5* ÷ P × 100, where S is the amount of input sample DNA in ng, P is the amount of input positive control in ng, and 5* is a factor to normalize 5-hmC in the positive control to 100%, as the positive control contains only 20% of 5-hmC.

### Chromatin accessibility assay

Accessibility of chromatin to gene promoters was assessed by nuclease-dependent chromatin degradation combined with qPCR using Epiquik Chromatin Accessibility Assay Kit (Epigentek, P-1047-48) according to the manufacturer’s instructions. Chromatin was isolated and digested from cells, followed by DNA purification. Chromatin preparations from each sample were subjected to either nuclease digestion (Nse mix) or no nuclease digestion (No Nse). DNA was amplified by qPCR using the LightCycler^®^ 480 II PCR (Roche, Indianapolis). Primers specific for target genes were utilized for the assay. All samples were validated using positive and negative control primer sets provided by the manufacturer. The degree of Ct shift between digested and undigested samples provided an indication about chromatin structure, where insignificant Ct shifts between digested and undigested samples represented DNA in the heterochromatin region that was inaccessible to the nucleases, whereas larger Ct shifts represented DNA in the euchromatin status that remained attainable to nucleases. Fold enrichment (FE) was calculated by the formula FE = 2(NseCT−no Nse CT)  × 100%.

### Immunohistochemistry

Breast cancer tissue microarray slides BR10010d and BR20837a (breast cancer and matched metastatic carcinoma tissue array) and BR487 (triple-negative breast cancer tissue array) were purchased from US Biomax, Inc. (Rockville, MD) and were processed for immunohistochemical staining for mdig and H3K9me3 proteins. Paraffin-embedded tissue sections were deparaffinized with xylene and hydrated in a series of alcohol gradients. To quench endogenous peroxidase activity, slides were incubated with 1.5 to 3% H_2_O_2_ in PBS for 20 min at room temperature. Heat-mediated antigen retrieval was performed by boiling tissue sections in citrate buffer with pH 6.0 for 20 min in a microwave. To block nonspecific binding of immunoglobulin, slides were incubated with a solution containing 5% goat serum, 0.2% Triton X-100 in PBS for 2 h at room temperature, followed by incubation with primary antibodies against mdig (mouse anti-MINA, Invitrogen with 1:50 dilution) and H3K9me3 (rabbit anti-H3K9me3, Abcam 8898 with 1:200 dilution) overnight at 4 °C. Goat anti-mouse and goat anti-rabbit biotinylated secondary antibodies were subsequently applied at 1:200 dilution and incubated for 2 h at room temperature. Slides were then incubated with ABC reagent (Vectastatin Elite ABC kit) for 45 min at room temperature, and the chromogen was developed with diaminobenzidine (DAB). Slides were counterstained with hematoxylin (Sigma-Aldrich, St. Louis, MO) and mounted with Entellan® (Electron Microscopy Sciences, Hatfield, PA). All incubation steps were carried out in a humidified chamber, and all washing steps were performed with 1 × PBS. Images were captured under bright field of a Nikon Eclipse Ti-S Inverted microscope (Mager Scientific, Dexter MI, USA) and analyzed using Nikon’s NIS Elements BR 3.2 software.

### Western blotting

Total cellular proteins were prepared by lysing cells via sonication in 1 × RIPA buffer (Millipore, Billerica, MA) supplemented with phosphatase/protease inhibitor cocktail and 1 mM PMSF. Lysed cells were then centrifuged and supernatant isolated as protein, which was quantified using the Micro BCA Protein Assay Reagent Kit (Thermo Scientific, Pittsburgh, PA). Prior to loading onto SDS–PAGE gels, samples were boiled in 4 × NuPage LDS sample buffer (Invitrogen) containing 1 mM dithiothreitol (DTT). Samples were run on 7.5%, 10% or 15% SDS-PAGE gels, and separated proteins were then transferred to methanol-wetted PVDF membranes (Invitrogen). Membranes were subsequently blocked in 5% nonfat milk in TBST and probed with the indicated primary antibodies at dilutions of 1:1000, 1:2000 or 1:5000 overnight at 4 °C. The next day, membranes were washed with TBST and incubated with horseradish peroxidase (HRP)-conjugated secondary antibodies at dilutions of 1:2000 or 1:5000 at room temperature for 1 h. Immunoreactive bands were visualized through SuperSignal™ West Pico Chemiluminescent Substrate detection system (Thermo Scientific, Rockford, IL). Mdig (mouse) antibody was purchased from Invitrogen. C-myc antibody was purchased from Santa Cruz Biotechnology (Dallas, Texas, USA). Antibodies for H3K9me, H3K9me2, H3K9me3, and total histone H3 were purchased from Abcam (Cambridge, MA). All secondary antibodies were purchased from Cell Signaling Technology (Danvers, MA, USA). All presented data are representative of at least three independent experiments.

### Data mining

We analyzed open-access breast cancer patient databases containing genomic and transcriptomic data from cBioportal, Oncomine and UCSC Xena web platforms. The association between mdig expression and DNA methylation was calculated using MEXPRESS. Visualization of TCGA data for mdig in invasive breast carcinoma was created based on MEXPRESS and USCS Xena, consisting of 871 and 1,247 cases, respectively. Only statistically significant results are reported.

### Statistical analysis

All cell culture experiments were performed in triplicate at minimum, and error bars are shown as ± S.D. Chi-square tests and Fisher’s exact tests were applied to analyze the relationship between mdig and clinicopathological parameters. A *P*-value of < 0.05 was considered statistically significant.

## Results

### Mdig is pro-proliferative for noncancerous breast cells

**Fig. 1:**
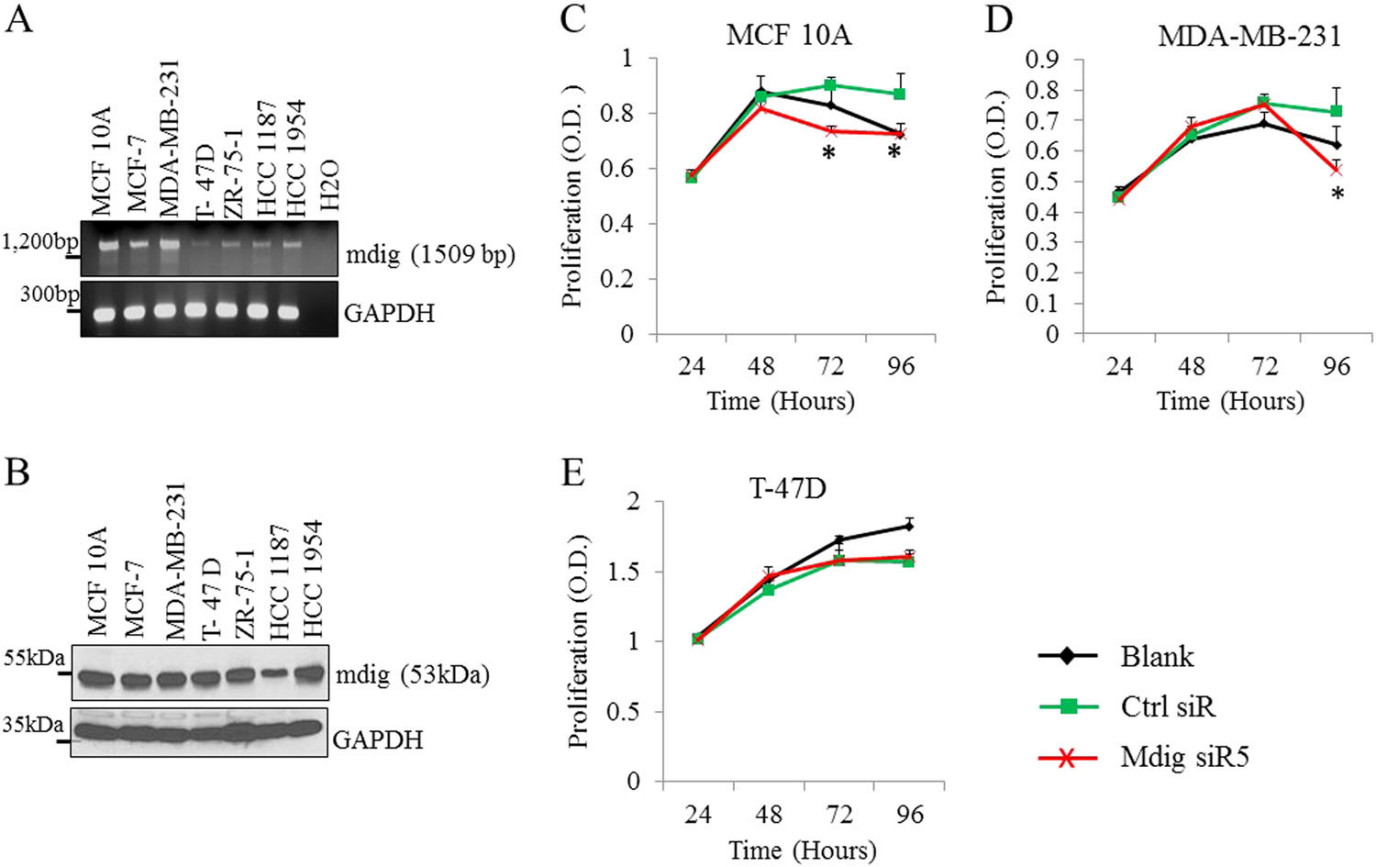
Mdig regulates cell proliferation. RT-PCR (**a**) and western blotting (**b**) show expression of mdig gene and protein, respectively, in MCF10A noncancerous human breast cells and breast cancer cell lines MCF-7, MDA-MB-231, T-47D, ZR-75-1, HCC 1187 and HCC 1954. **c**, **d** and E, MTT assays of indicated cells for 24, 48, 72, and 96 h post transfection with control siRNA (Ctrl siR) or a siRNA targeting mdig, mdig siR5 (*p < 0.05, *n* = 3)

To determine basal levels of mdig expression, we measured expression of mdig at the mRNA and protein levels in the noncancerous mammary epithelial cell line MCF10A and six breast cancer cell lines. As depicted in Fig. 1, mdig is clearly expressed in both noncancerous breast and breast cancer cells (Fig. 1a, b). As mdig already exhibits basal expression in all of these cells, we next depleted mdig levels using two different siRNAs, mdig siR2 and mdig siR5, respectively, to investigate the role of mdig on cell growth in MCF10A, MDA-MB-231 and T-47D cells. A significant decrease in cell proliferation was noted in MCF10A cells, but not in MDA-MB-231 and T-47D cells, following mdig silencing (Fig. 1c–e). In fact, in the MDA-MB-231 breast cancer cell line, a marginal increase in cell growth was observed 72 h after the silencing of mdig (Fig. 1d). These results indicate that mdig promotes proliferation in noncancerous mammary epithelial cells but not in breast cancer cells.

### Silencing mdig enhances migration and invasion of breast cancer cells

**Fig. 2:**
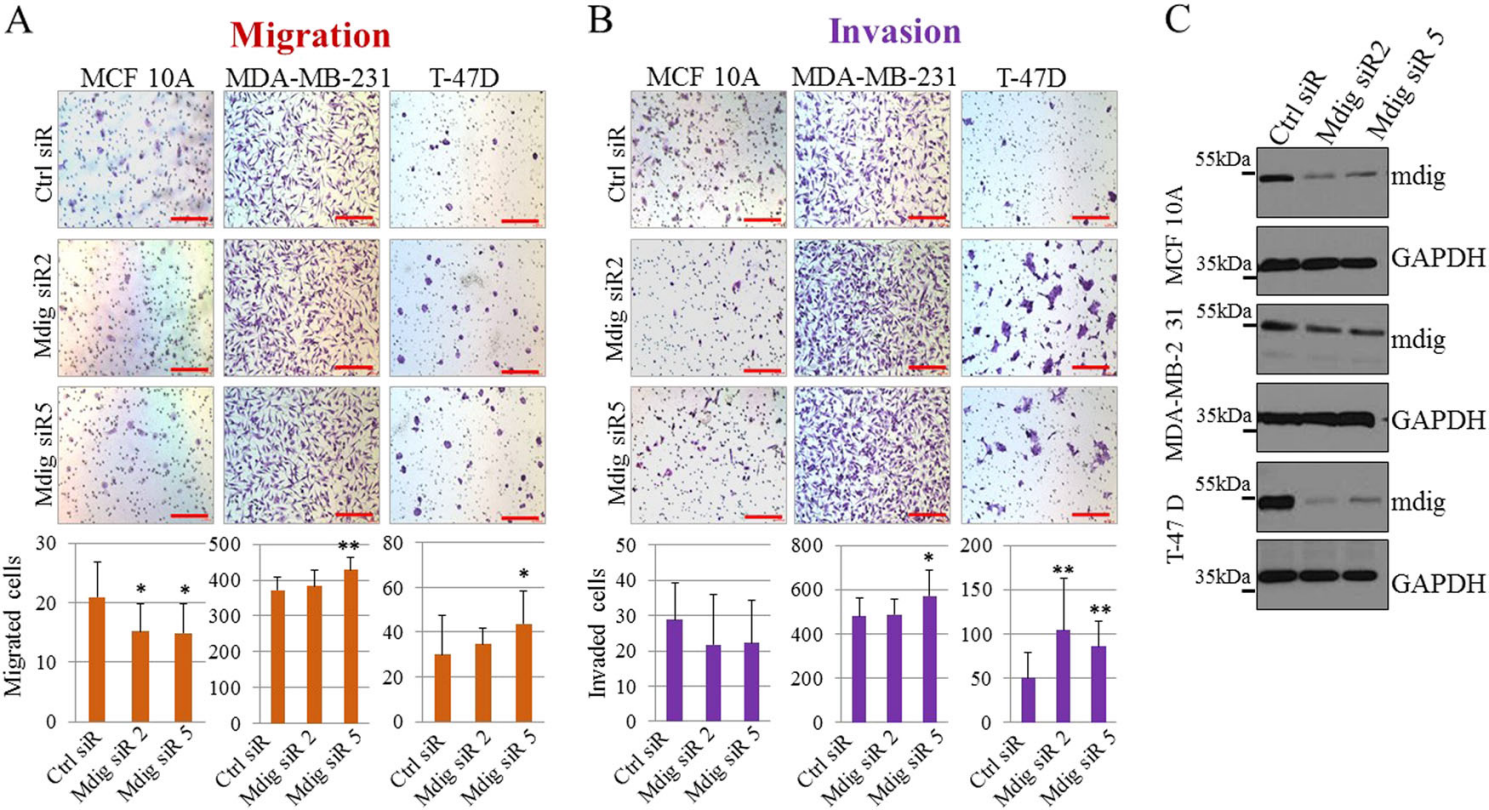
Mdig affects cell motility and invasion of noncancerous breast and breast cancer cells. Transwell migration (**a**) and Matrigel invasion (**b**) assay of indicated cell lines following transfection with Ctrl siRNA, mdig siR2 or mdig siR5 24 h post transfection. Scale bar = 200 μM. Quantification of number of migrated cells and invaded cells depicted by a bar graph below each of the figure panels. Ten randomly selected fields were counted for migrated and invasive cells (*n* = 10). * p < 0.05; **p < 0.01. **c**. Western blotting shows a decrease in mdig protein for indicated cell lines 24 h after knockdown of mdig by siRNAs

We utilized transwell migration and Matrigel invasion assays to determine the influence of mdig on cell motility and invasion. Noncancerous MCF10A cells exhibit some characteristics of cell motility and invasion.^15,16^ Therefore, in this assay, we included MCF10A cells for comparison with breast cancer cells. Although statistically marginal, silencing mdig appears to reduce the migration of MCF10A cells (Fig. 2a). In contrast, in two cancer cell lines, MDA-MB-231 and T-47D, silencing mdig resulted in a measurable increase in migration, particularly in cells transfected with mdig siRNA 5 (Fig. 2a). Invasion assays showed notably enhanced cell invasion of MDA-MB-231 and T-47D cells after mdig silencing, but not in MCF10A cells (Fig. 2b), despite a similar silencing effect being achieved among these three cell lines (Fig. 2c).

### Differential regulation of mdig on cell motility genes and invasion between noncancerous breast and breast cancer cells

**Fig. 3:**
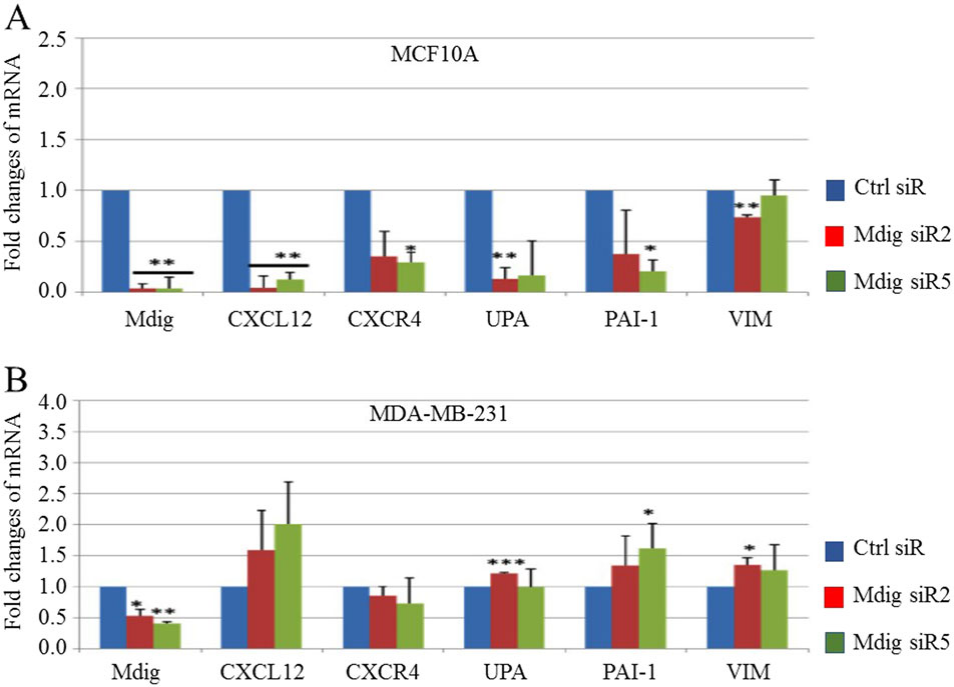
Mdig regulates expression of genes implicated in cell motility and invasiveness of breast cancer. Real time PCR for determination of genes involved in cell motility and invasion in MCF10A noncancerous breast cells (upper panel) and MDA-MB-231 breast cancer cells (lower panel) after transfection with indicated siRNAs. Fold changes were calculated relative to Ctrl siR and the housekeeping gene GAPDH. Data represents ± S.D., *n* = 3

Prompted by the differential role of mdig in cell growth and migration/invasion between noncancerous breast and breast cancer cells, we next investigated the expression levels of genes that are critically involved in the motility, invasion and metastasis of breast cancer cells. These genes include the extracellular matrix-degrading protease urokinase plasminogen activator (UPA) and its inhibitor plasminogen activator inhibitor-1 (PAI-1), CXCR4 and its ligand CXCL12, and an epithelial-mesenchymal transition (EMT) marker, vimentin (VIM). Mdig silencing in noncancerous MCF10A breast cells significantly reduced the expression of genes important for invasion and migration, as well as for the EMT process (Fig. 3a). In contrast, in MDA-MB-231 breast cancer cells, siRNA depletion of mdig elevated the expression of these genes (Fig. 3b). These results further support the observed differential role of mdig silencing on cell migration and invasion between noncancerous and breast cancer cells (Fig. 2).

### Mdig regulates histone and DNA methylation

**Fig. 4:**
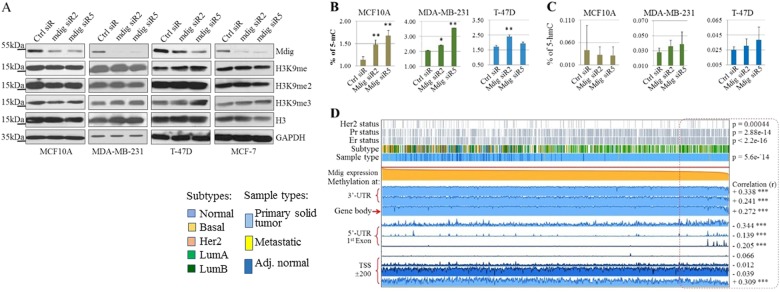
Mdig silencing enhances histone and DNA methylation. **a** Protein levels of mdig, H3K9me, H3K9me2, H3K9me3, H3, and GAPDH were determined by western blotting in the indicated cell lines after transfection with indicated siRNAs. **b**, **c** Total DNA methylation in indicated cell lines transfected with siRNAs were determined by measuring 5-methylcytosine (5-mC, B) and 5-hydroxymethycytosine (5-hmC, C) as indicated in the Materials and Methods. **d** Visualization of TCGA data containing 871 cases of invasive breast cancer for expression and DNA methylation of mdig genes using MEXPRESS (http://mexpress.be/). Samples are ordered by mdig expression values

Since mdig had been implicated in regulating ribosomal protein hydroxylation and histone methylation,^3,17^ as well as DNA and histone methylation in cancer cell metastasis,^18^ we subsequently evaluated histone H3 lysine 9 (H3K9) methylation and DNA methylation in noncancerous breast and breast cancer cells. In MCF10A noncancerous breast cells, despite significant silencing of mdig by two different siRNAs, no changes were observed in mono-, di- or tri-methylation of H3K9 (H3K9me1, H3K9me2, and H3K9me3). In MDA-MB-231, T-47D, and MCF-7 breast cancer cell lines, a marginal increase of H3K9me3 was noted in cells transfected with mdig siRNAs (Fig. 4a). In T-47D cells, mdig silencing also resulted in notably increased H3K9me1.

To determine the possible role of mdig in DNA methylation, we also measured global DNA methylation by assessing 5-methylcytosine (5mC) and 5-hydroxymethylcytosine (5hmC) in cells transfected with mdig siRNAs. Unexpectedly, a significant increase in 5mC was noted in both noncancerous MCF10A cells and two cancer cell lines, MDA-MB-231 and T-47D, following mdig silencing (Fig. 4b). There was no significant change in 5hmC, an intermediary of DNA demethylation from 5mC, observed in cells with mdig silencing (Fig. 4c), possibly due to the limitation of the sensitivity of the method used to detect the lower levels of 5hmC in these cells. Since DNA methylation determines the degrees of gene expression, we also looked at the relationship between mdig expression and DNA methylation of the mdig gene itself in The Cancer Genome Atlas (TCGA) database, containing information on 871 cases of invasive breast carcinoma. Depending on the regions where DNA methylation occurs, it can both positively and negatively influence expression of the mdig gene (Fig. 4d). In general, DNA methylation at the 3′-UTR, gene body and regions encompassing the transcription start site (TSS) correlates with increased expression of mdig. In contrast, methylation at the 5′-UTR and the first exon strongly correlates with the downregulation of mdig expression among these tumor samples (enclosed in a red dash-lined box, Fig. 4d). These data, thus, clearly indicate that the loss of mdig favors DNA methylation, or in other words, mdig acts as a DNA demethylase or a cofactor in assisting DNA demethylation. Reducing mdig by siRNA, therefore, results in the accumulation of methylated cytosine in the DNA of cells. Negative regulation of DNA methylation on gene expression is most likely determined by methylation in the 5′-UTR and/or the first exon region, as shown by the mdig gene.

Interestingly, we also noted a clear correlation among subtypes of breast cancer, DNA methylation and mdig expression in breast cancer. As depicted in Fig. 4d, decreased mdig expression and elevated 5′-UTR/1^st^ Exon DNA methylation of the mdig gene are mostly associated with Her2-negative, PR- and ER-positive breast cancers. Although statistically insignificant, luminal A breast cancer exhibited lower levels of mdig expression.

### Mdig influences chromatin accessibility for genes involved in migration and invasion

**Fig. 5:**
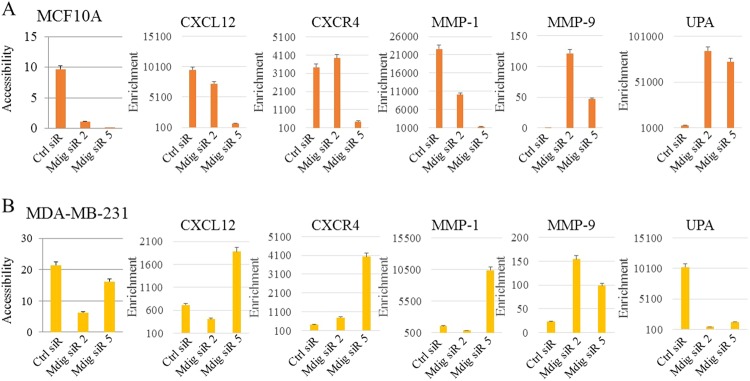
Mdig regulates chromatin accessibility. **a**, **b** Chromatin accessibility assay coupled with real time PCR for the indicated genes were performed in MCF10A (**a**) and MDA-MB-231 (**b**) with or without mdig silencing. Fold enrichment was calculated using the formula FE = 2ˆ(NseCT-noNseCT) × 100%. Error bars = percentage error of value 5

It has been well established that methylation of DNA and histones alters the accessibility of chromatin through the formation of euchromatin or heterochromatin. Considering the effects of mdig on both DNA and histone methylation, we next evaluated chromatin accessibility following mdig silencing. Chromatin accessibility is determined by mapping nucleosome positions along the genome because if the chromatin is more condensed due to the formation of heterochromatin, the DNA becomes less accessible to transcription factors and other DNA binding proteins. In contrast, if the chromatin is more open, i.e., the formation of euchromatin, the DNA is more accessible, and hence, surrounding genes are actively transcribed. Analyzing the DNA of noncancerous MCF10A cells and breast cancer MDA-MB-231 cells in response to mdig knockdown revealed that loss of mdig reduces the accessibility of the genome, suggesting that mdig silencing favors the formation of heterochromatin (Fig. 5a, b). However, there was a significant difference between noncancerous cells and cancer cells in the chromatin accessibility of individual genes involved in migration and invasion. In MCF10A cells, a loss of mdig favored the closed chromatin conformation (heterochromatin) of the genome for metastatic genes CXCL12, CXCR4, and MMP-1, while promoting open chromatin (euchromatin) structures for the EMT genes MMP-9 and UPA (Fig. 5a). Strikingly, in MDA-MB-231 breast cancer cells, the loss of mdig appeared to favor the open chromatin structure of the genome for metastatic genes CXCL12, CXCR4, MMP-1, and MMP-9, while enhancing the formation of closed chromatin for UPA (Fig. 5b).

### Loss of mdig expression in invasive and late stage breast cancer

**Fig. 6:**
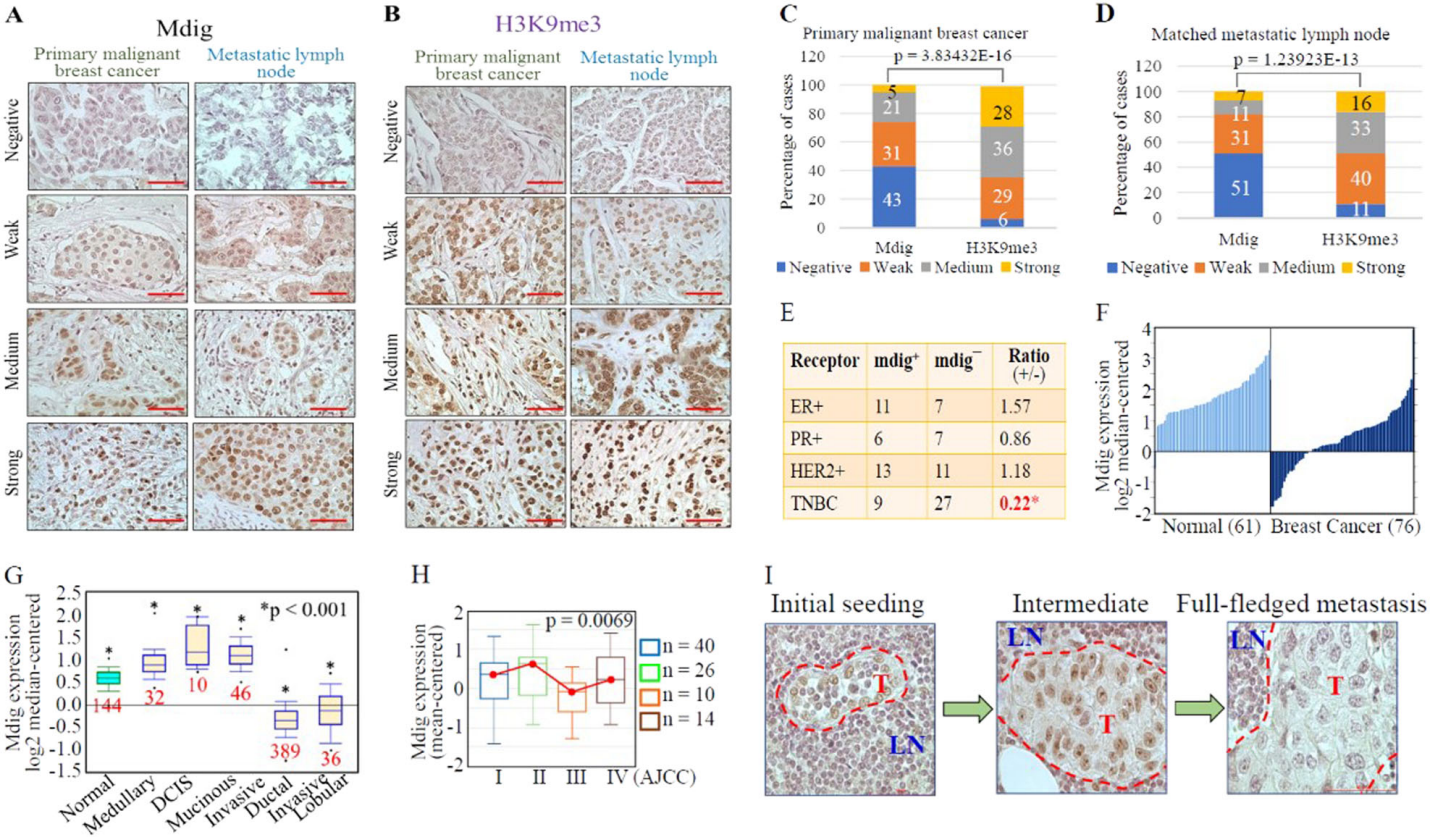
Loss of mdig expression in invasive and metastatic breast cancers. **a** Mdig expression pattern in primary malignant breast carcinoma and matched metastatic carcinomas in lymph nodes. **b** H3K9me3 staining pattern in primary malignant breast carcinoma and matched metastatic carcinoma in lymph nodes. Data represent 154 cases, ×40 magnification, scale bar = 50 µm. **c**, **d** Quantification of scored images for mdig and H3K9me3 in breast cancer (**c**) and matched metastatic lymph nodes (D), respectively. **e**, **f** Loss of mdig expression in TNBC and invasive breast cancer (data source: http://www.oncomine.org). **g** Relative levels of mdig expression in breast cancers with different histological subtypes (CURTIS Breast, *n* = 2136, Oncomine). **h** Relative levels of mdig expression in breast cancers with different AJCC stages (UCSC Xena, http://xena.ucsc.edu). **i** Loss of mdig expression in breast cancer cells that metastasized to lymph nodes. Representative images from the metastatic lymph nodes showing the characteristic phenotype of cells positive for mdig. Left panel, very few tumor cells that are positive for mdig were detected in lymph nodes; middle panel, increased number of tumor cells in the lymph nodes, some of which showed weak staining for mdig; right panel, full-fledged tumor within the lymph node showing loss of mdig in tumor cells. LN = lymph node area, T = Tumor area; ×40 magnification. Scale bar = 50 µm

Histone modifications play crucial roles in organizing the nuclear architecture and regulating the transcription of several genes implicated in breast carcinogenesis.^19^ Prompted by the observation that reduced mdig expression enhances the chromatin accessibility of genes implicated in metastasis, we queried the expression status of mdig, as well as levels of the heterochromatin marker H3K9me3, in breast cancer patients. We utilized a human tissue microarray panel consisting of 154 primary malignant breast tumors along with matched metastatic lymph nodes. By scoring the staining intensities of mdig and H3K9me3 using ImageJ software, both primary tumors and matched metastatic lymph nodes showed negative, weak, medium, and strong signals for mdig and H3K9me3 (Fig. 6a, b). When comparing staining scores, an inverse relationship was revealed between mdig and H3K9me3 in both primary tumors and matched metastatic lymph nodes (Fig. 6c, d), consistent with the observed enhancement of H3K9me3 among breast cancer cells in response to mdig silencing (Fig. 4a).

Further evaluation of mdig levels in relation to the clinicopathological parameters of patient samples revealed that mdig expression status is significantly correlated with molecular subtypes, particularly ER, PR, and HER2 status. A significant reduction of the mdig positive ratio was noted among samples of triple-negative breast cancer (TNBC), the most aggressive, and possibly the most metastatic, breast cancer (Fig. 6e). Loss of mdig expression in TNBC was also confirmed in an RNA-sequence (RNA-seq) database containing 42 cases of TNBC compared to 21 adjacent normal breast tissues. Among three full-length mdig transcripts, two showed a significant reduction in TNBC tumors relative to adjacent normal breast tissue (http://syslob4.nchu.edu.tw/CRN, data not shown). To further support this notion, we analyzed several TCGA datasets through a data-mining platform and found that levels of mdig expression varied depending on the histological type and disease stage. When comparing mdig expression in all breast cancers with normal breast tissues, reduced mdig expression was noted in cancers (Fig. 6f). However, when cancers were stratified based on histological subtypes, cancers with less metastatic potential, such as medullary carcinoma, ductal carcinoma in situ (DCIS), and mucinous carcinoma, exhibited increased expression of mdig, whereas invasive ductal and lobular carcinoma showed significantly decreased mdig expression (Fig. 6g). Among the different stages of breast cancer, in general, stages III and IV showed reduced mdig expression relative to stages I and II breast cancers, suggesting a loss of mdig expression in later-stage breast cancers (Fig. 6h). These data clearly indicate that mdig expression in breast cancer is context dependent. During initiating or earlier stages, mdig expression is higher. However, mdig expression decreases in aggressive or later-stage breast cancers, such as TNBC and cancers that tend to be invasive and metastatic.

### Decreased mdig expression in later stage lymph node metastasis

Since we observed a significant reduction of mdig expression in TNBC and other invasive breast cancers (Fig. 6e–g), we assessed the expression pattern of mdig in breast cancer cells that metastasized to the lymph nodes at different stages. Careful analysis of histopathological images of lymph nodes after staining for mdig revealed three distinct patterns of mdig expression. During the initial seeding stage when very few breast cancer cells had colonized the lymph nodes, tumor cells were strongly positive for mdig staining (Fig. 6i, left panel). In intermediate-stage lymph node metastasis of the breast cancer cells, some tumor cells retained mdig expression, while others showed scarce mdig staining (Fig. 6i, middle panel). Lastly, in full-fledged stages where the metastatic lesions of tumor cells had advanced to almost the entire area of the lymph node, leaving very few lymph cells at the metastasized tumor periphery, tumor cells were found to be completely negative for mdig expression (Fig. 6i, right panel). Accordingly, we hypothesize that mdig expression is lost in advanced stages of metastasis.

## Discussion

Breast cancer is a complex disease with several different histological and molecular subtypes.^20^ Most breast tumors originate from the milk ducts or milk-producing lobules. Based on the invasiveness and metastatic behaviors of the tumors, breast cancers can be further classified into noninvasive and invasive carcinomas. Ductal carcinoma in situ (DCIS), for example, is viewed as a noninvasive or early stage breast cancer due to its confined location in the milk ducts that does not spread into neighboring breast tissue or lymph nodes. In general, approximately 80% of breast cancers are invasive ductal carcinoma (IDC). Similarly, most triple-negative breast cancers (TNBC) are basal cell-like carcinoma in the ductal area and are highly metastatic.^21^ However, some types of IDCs, such as medullary and mucinous carcinomas, are less likely to metastasize to the lymph nodes than other types of IDCs. Accordingly, the prognosis of breast cancer patients are largely dependent upon the metastatic status of the tumors.

We previously demonstrated that expression levels of mdig, also known as mina53, NO52, JMJD10, and RIOX2, predicts survival outcomes of breast cancer patients, depending on the status of lymph node metastasis.^22^ For breast cancers without lymph node metastasis, higher levels of mdig predict poorer overall survival of breast cancer patients. In contrast, higher levels of mdig predict better overall survival of breast cancer patients who have lymph node metastasis. To decipher the mechanism behind the paradoxical prognostic values of mdig in breast cancers, the present study demonstrated that mdig negatively regulates DNA methylation and H3K9me3 in cancer cells and tissues, which influences the accessibility of chromatin and the expression of genes involved in cancer cell migration and invasion. A detailed analysis clearly demonstrates increased expression of mdig in early stages of breast cancers and in noninvasive breast cancers, including medullary carcinoma, DCIS and mucinous carcinoma (Fig. 6g, h). In contrast, mdig expression is lost in the invasive carcinoma TNBC and in cancer cells that metastasized to lymph nodes.

The molecular function of mdig remains to be fully elucidated. Given the presence of a conserved JmjC domain in its amino acid sequence, mdig was first assumed to be a histone demethylase.^4^ However, both cellular experiments and test tube reactions revealed that mdig has very marginal histone demethylase activity toward H3K9me3.^9,23^ Even in breast cancer cells presented in this report, silencing mdig only achieved limited enhancement of H3K9me3. Structural characterization of the JmjC domain of mdig further suggests that this protein is a ribosomal protein hydroxylase rather than a histone demethylase,^17,24^ explaining the weak demethylation activity observed on H3K9me3 in cellular experiments. Nevertheless, studies in human cancers still suggest involvement of mdig in H3K9me3 demethylation. In human lung cancer, we observed a clear inverse relationship between levels of mdig and H3K9me3.^4^ This notion was further confirmed in human breast cancers as reported here (Fig. 6). In glioblastoma cells, mdig knockdown by shRNAs not only increased levels of H3K9me3 but also inhibited expression of several cell cycle regulatory proteins, such as cyclin B, cyclin D1, CDK1, CDK2, etc.^25^ Similarly, mdig appears to be highly capable of reducing levels of H3K9me3 in human hepatocellular carcinoma (HCC).^26^ Increased expression of mdig was observed in approximately 70% of HCC samples collected from 155 patients. In HCC cell lines, overexpression of mdig decreased, whereas silencing mdig by shRNAs increased the level of H3K9me3. In addition to H3K9me3, our recent CRISPR-Cas9-based gene knockout studies suggest that mdig may contribute to demethylation of H3K27me3, but not H3K4me3 or H3K36me3, in human bronchial epithelial cells (Zhang et al, unpublished observations).

The unexpected finding from this report is the pronounced effect of mdig on DNA demethylation. In both noncancerous breast and breast cancer cells, mdig silencing resulted in a significant increase of DNA methylation (Fig. 4), indicating that mdig is somehow involved in DNA demethylation. The most studied DNA demethylases, the TET family DNA hydroxylases, remove the methyl group from 5-methylcytosine (5mC) through hydroxylation of the methyl group to form 5-hydroxymethylcytosine (5hmC).^27^ Interestingly, mdig has been shown to be a hydroxylase that is able to hydroxylate His-39 on the 60 S ribosomal protein L27a (Rpl27a).^17,24^ Thus, it was worthwhile to examine whether mdig hydroxylates 5mC directly. An additional possibility is that mdig contributes to DNA demethylation through regulating the co-factors important for DNA methylation. We demonstrated that mdig is highly capable of inducing expression of H19, a large intergenic noncoding RNA.^23^ Some recent studies suggest that H19 binds to and inhibits S-adenosylhomocysteine hydrolase (SAHH), leading to accumulation of S-adenosylhomocysteine (SAH), a potent feedback inhibitor of S-adenosylmethionine (SAM)-dependent DNA methylation.^28,29^ Furthermore, mdig may also facilitate demethylation efficiency through authentic DNA demethylases or other pathways essential for DNA demethylation. Indeed, we have demonstrated physical interaction between mdig and a number of proteins critical for DNA replication and DNA damage repair,^30^ many of which are also actively or passively involved in DNA demethylation.

**Fig. 7:**
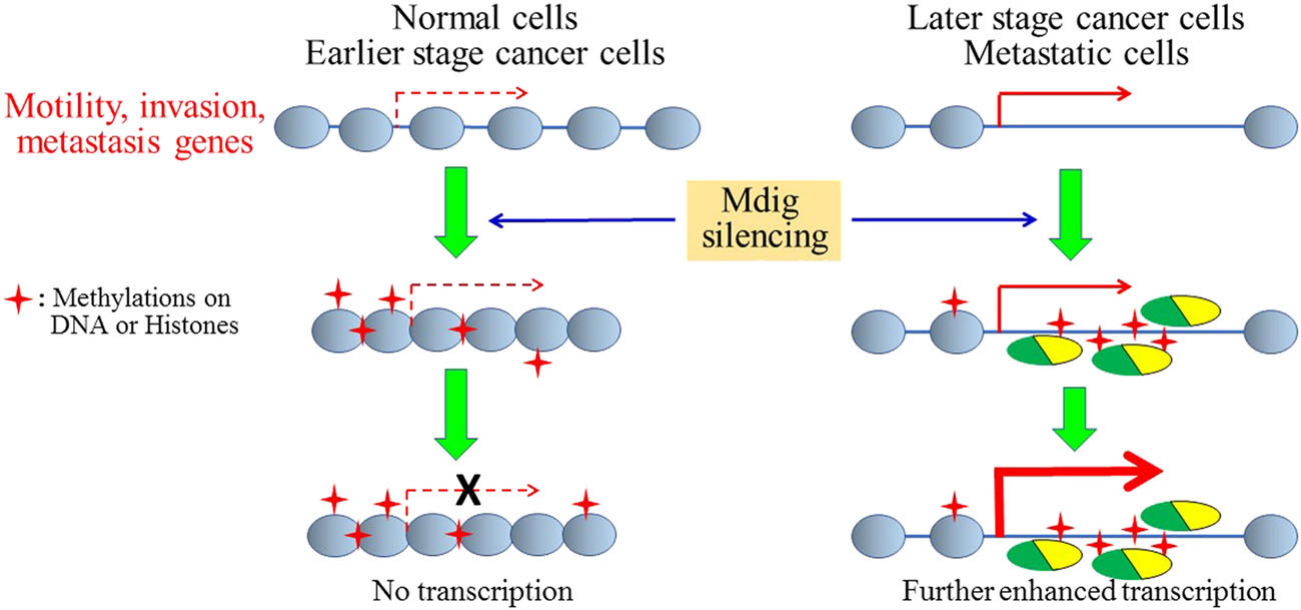
Schematic diagram showing opposing effects of mdig silencing on expression of genes for cell motility and invasion between earlier and later stage cancer cells. In normal cells or earlier stage cancer cells, the chromatin of these genes is partially open. Mdig silencing increases both H3K9me3 and DNA methylation, causing further condensation of the chromatin and inhibition of these genes. In later stage and metastatic cancer cells, the chromatin configuration of these genes is widely open. Mdig silencing enhances DNA and histone methylation in the intron or gene body of these genes, and a few of the nucleosome histone proteins, which may result in recruitment of transcriptional regulatory proteins for enhanced transcription of genes involved in cell motility, invasion and metastasis

Both DNA and H3K9me3 are factors determining the configuration and accessibility of chromatin. Silencing mdig resulted in increased H3K9me3 and DNA methylation, which should reduce the accessibility of chromatin. This appears to be true in both noncancerous breast and breast cancer cells, where a decrease of overall chromatin accessibility was noted following mdig silencing (Fig. 5). However, for accessibility of individual genes involved in cell motility and EMT, an opposing effect was observed in response to mdig silencing on expression and chromatin accessibility of these genes between noncancerous and cancer cells (Figs. 3 and 5). In MCF10A noncancerous breast cells, mdig silencing reduced chromatin accessibility of the majority of genes in cell motility and invasion, except MMP-9 and UPA. In MDA-MB-231 cells, a TNBC cell line, despite mdig silencing reducing overall chromatin accessibility, accessibility of genes for cell motility and invasion was increased, except for UPA. Thus, it is possible that the effect of mdig on chromatin accessibility of individual genes may depend on preexisting chromatin status. When the chromatin of individual motility/invasion genes are in a partial opening status in normal or noncancerous cells, silencing mdig elevates H3K9me3 and DNA methylation, causing tight compaction of chromatin and inhibiting expression of these genes. In cells from later stage tumors, due to the preopening status of chromatin, increased levels of H3K9me3 and DNA methylation resulted from mdig silencing may most likely occur in the gene body or intronic regions of these genes, leading to recruitment of additional transcriptional regulators to promote expression of these genes further (Fig. 7). This could also explain why DNA methylation in the gene body was actually correlated to increased expression of mdig in some breast cancer samples (Fig. 4d).

In summary, the present study unraveled a previously undefined paradoxical role for mdig in breast cancer. Mdig is oncogenic in noncancerous cells by promoting cell growth or cell cycle transition. In cancer cells, particularly in later stage tumors, mdig is tumor suppressive through negatively regulating the migration, invasion and metastasis of cancer cells. It is plausible, therefore, to speculate a stage- or context-dependent role of mdig in the development of breast cancer. Mdig expression levels may be higher in initiating or earlier stages of breast cancer, which may be essential for tumor growth. In later stage tumors, mdig expression is suppressed, which favors invasion and metastasis of tumor cells. The dual role of mdig in breast cancer was supported by an earlier observation showing that the oncogene c-myc, a possible transcriptional regulator for mdig,^2^ can be both oncogenic for cancer cell proliferation and suppressive for cancer cell motility, invasion and metastasis,^31^ In human neuroblastoma, Schwab et al.^32^ also provided evidence showing that N-myc, a myc family member, may play an important role in tumor regression, leading to favorable outcomes for some later stage neuroblastomas. It is currently unknown whether the paradoxical role of mdig occurs in breast cancer only or in other cancers as well. In human lung cancer, we observed differential prognostic values for mdig in patients with AJCC stages N0, N1 and N2,^9^ suggesting a similar effect in both breast cancer and lung cancer. Taken together, the findings in the present report provide an explanation for the discrepancies of function, expression and prognostic power of mdig in human breast cancer. These findings may be important in the development of therapeutics that target mdig in breast cancer or other tumors.

## References

[1] Zhang Y (2005). The Human mineral dust-induced gene, mdig, is a cell growth regulating gene associated with lung cancer. Oncogene.

[2] Tsuneoka M, Koda Y, Soejima M, Teye K, Kimura H (2002). A novel myc target gene, mina53, that is involved in cell proliferation. J. Biol. Chem..

[3] Thakur C, Chen F (2015). Current understanding of mdig/MINA in human cancers. Genes Cancer.

[4] Lu Y (2009). Lung cancer-associated JmjC domain protein mdig suppresses formation of tri-methyl lysine 9 of histone H3. Cell Cycle.

[5] Mori T (2013). Ablation of Mina53 in mice reduces allergic response in the airways. Cell Struct. Funct..

[6] Thakur C (2015). Oncoprotein mdig contributes to silica-induced pulmonary fibrosis by altering balance between Th17 and Treg T cells. Oncotarget.

[7] Hemmers S, Mowen KA (2009). T(H)2 bias: Mina tips the balance. Nat. Immunol..

[8] Yosef N (2013). Dynamic regulatory network controlling TH17 cell differentiation. Nature.

[9] Yu M (2014). Paradoxical roles of mineral dust induced gene on cell proliferation and migration/invasion. PLoS ONE.

[10] Sun J (2014). Carcinogenic metalloid arsenic induces expression of mdig oncogene through JNK and STAT3 activation. Cancer Lett..

[11] Esteller M (2008). Epigenetics in cancer. New Eng. J. Med..

[12] Lustberg MB, Ramaswamy B (2011). Epigenetic therapy in breast cancer. Curr. Breast Cancer Rep..

[13] Esteller M (2000). Promoter hypermethylation and BRCA1 inactivation in sporadic breast and ovarian tumors. J. Natl. Cancer Inst..

[14] Radpour R (2011). Integrated epigenetics of human breast cancer: synoptic investigation of targeted genes, microRNAs and proteins upon demethylation treatment. PLoS ONE.

[15] Bessette DC (2015). Using the MCF10A/MCF10CA1a breast cancer progression cell line model to investigate the effect of active, mutant forms of EGFR in breast cancer development and treatment using gefitinib. PLoS ONE.

[16] Zhang X (2015). Notch1 induces epithelial-mesenchymal transition and the cancer stem cell phenotype in breast cancer cells and STAT3 plays a key role. Int. J. Oncol..

[17] Chowdhury R (2014). Ribosomal oxygenases are structurally conserved from prokaryotes to humans. Nature.

[18] Sahin M, Sahin E, Gumuslu S, Erdogan A, Gultekin M (2010). DNA methylation or histone modification status in metastasis and angiogenesis-related genes: a new hypothesis on usage of DNMT inhibitors and S-adenosylmethionine for genome stability. Cancer Metastas-. Rev..

[19] Hon GC (2012). Global DNA hypomethylation coupled to repressive chromatin domain formation and gene silencing in breast cancer. Genome Res..

[20] Hsu JL, Hung MC (2016). The role of HER2, EGFR, and other receptor tyrosine kinases in breast cancer. Cancer Metastas-. Rev..

[21] Bianchini G, Balko JM, Mayer IA, Sanders ME, Gianni L (2016). Triple-negative breast cancer: challenges and opportunities of a heterogeneous disease. Nat. Rev. Clin. Oncol..

[22] Thakur C (2014). Increased expression of mdig predicts poorer survival of the breast cancer patients. Gene.

[23] Chen B (2013). Mdig de-represses H19 large intergenic non-coding RNA (lincRNA) by down-regulating H3K9me3 and heterochromatin. Oncotarget.

[24] Ge W (2012). Oxygenase-catalyzed ribosome hydroxylation occurs in prokaryotes and humans. Nat. Chem. Biol..

[25] Huang MY, Xuan F, Liu W, Cui HJ (2017). MINA controls proliferation and tumorigenesis of glioblastoma by epigenetically regulating cyclins and CDKs via H3K9me3 demethylation. Oncogene.

[26] Huo Q (2017). Dysfunction of IKZF1/MYC/MDIG axis contributes to liver cancer progression through regulating H3K9me3/p21 activity. Cell Death Dis..

[27] Wu SC, Zhang Y (2010). Active DNA demethylation: many roads lead to Rome. Nat. Rev. Mol. Cell Biol..

[28] Zhou J (2015). H19 lncRNA alters DNA methylation genome wide by regulating S-adenosylhomocysteine hydrolase. Nat. Commun..

[29] Zhong T (2017). Metformin alters DNA methylation genome-wide via the H19/SAHH axis. Oncogene.

[30] Wang W (2015). The proteomic investigation reveals interaction of mdig protein with the machinery of DNA double-strand break repair. Oncotarget.

[31] Liu H (2012). MYC suppresses cancer metastasis by direct transcriptional silencing of alphav and beta3 integrin subunits. Nat. Cell Biol..

[32] Westermann F (2008). Distinct transcriptional MYCN/c-MYC activities are associated with spontaneous regression or malignant progression in neuroblastomas. Genome Biol..

